# The Longitudinal Superdiffusive Motion of Block Copolymer in a Tight Nanopore

**DOI:** 10.3390/polym12122931

**Published:** 2020-12-08

**Authors:** Waldemar Nowicki

**Affiliations:** Faculty of Chemistry, Adam Mickiewicz University in Poznań, ul. Uniwersytetu Poznańskiego 8, 61-614 Poznań, Poland; gwnow@amu.edu.pl

**Keywords:** diffusion, lattice model, Monte Carlo method

## Abstract

The structure and dynamic properties of polymer chains in a confined environment were studied by means of the Monte Carlo method. The studied chains were represented by coarse-grained models and embedded into a simple 3D cubic lattice. The chains stood for two-block linear copolymers of different energy of bead–bead interactions. Their behavior was studied in a nanotube formed by four impenetrable surfaces. The long-time unidirectional motion of the chain in the tight nanopore was found to be correlated with the orientation of both parts of the copolymer along the length of the nanopore. A possible mechanism of the anomalous diffusion was proposed on the basis of thermodynamics of the system, more precisely on the free energy barrier of the swapping of positions of both parts of the chain and the impulse of temporary forces induced by variation of the chain conformation. The mean bead and the mass center autocorrelation functions were examined. While the former function behaves classically, the latter indicates the period of time of superdiffusive motion similar to the ballistic motion with the autocorrelation function scaling with the exponent *t*^5/3^. A distribution of periods of time of chain diffusion between swapping events was found and discussed. The influence of the nanotube width and the chain length on the polymer diffusivity was studied.

## 1. Introduction

In most cases, the self-diffusion process can be described as a relation between the mean square displacement (MSD) and time, expressed as a scaling law with the exponent equal to 1. However, in some systems, this simple relationship should be formulated in a more general way, as follows [1]:

(1) $$\langle r{\left(t\right)}^{2}\rangle \sim t^{v}$$

Depending on the value of the anomalous diffusion exponent, *v*, a subdiffusion (0 < *v* < 1) or superdiffusion (*v* > 1) can be distinguished [2,3,4]. The origin of the discrepancy between the properties of the system, and the scaling law with the exponent 1 is that its derivation requires that the Brownian particle should move in an infinite structureless medium. This assumption is generally incorrect when the Brownian motion takes place in a complex medium or when the diffusively migrating objects should be treated as structured species whose structural elements’ motions are only partly independent, but mutually correlated in general. Moreover, Equation (1) describes self-diffusion in a long period of time only. 

The subdiffusion deviations from the simple scaling exponent *v* = 1 have been predicted even for simple stochastic chains. According to the Rouse theory [5,6], the MSD of the beads forming the polymer chain increases with time in three time regimes: in the first and the last regime, the MSD scales with *v* = 1 (but with two different diffusion coefficients), whereas, between these two diffusional asymptotes, the MSD increases as the power of *t*^1/2^. The diffusion of the theta chain (Zimm model [6,7]) behaves similarly: The short, intermediate, and longtime MSD vs. time dependencies are power laws with exponents *v* = 1, 2/3, and 1, respectively. The deviations of *v* values predicted by the reptation theory are similar.

At very short time of self-diffusion, Brownian motion occurs in three basic regimes: (i) a ballistic-like motion before any molecular collisions [8]; (ii) a motion affected by hydrodynamic forces of the fluid [9], which begins when the moving object interacts with fluid molecules; and (iii) the actual random Brownian motion fractal in nature. The motion in the last regime—the random Brownian motion—causes the MSD values to increase linearly with time. The motion in the first regime—the free unidirectional ballistic motion scaling with *t*^2^—cannot last for long and occurs at timescales once deemed immeasurably small by Einstein [10]. 

The ballistic motion can be met not only in the transport of electrons in conductors, phonons in solids, and photons in opaque media but also in disordered liquids, colloids, bubbles, grains, and so forth that are on the verge of jamming [11]. This particular motion was observed for extended periods of time for particles in the air as a result of low viscosity of dispersion medium [5,12] and in short times in liquid water as a higher-density medium [10]. 

The transition between ballistic and diffusive motion is highly dependent on the properties and structure of a particular liquid [13]. Unusually long ballistic motions have been reported by research groups studying particles in polymer melts [14,15,16], polymer-grafted gold nanoparticles [17], polymer rings [18], DNA [19], enzymes [20], and even bacteria [21]. 

Dissolved polymer molecules located in the confined environment show unique behavior different from those of free macromolecules in the bulk phase [22]. The nanoconfinement alters not only the statics [23,24] but also the Brownian dynamics of a polymer chain [25,26]. The specific behavior of macromolecules results from a decrease in conformational entropy of macromolecules due to the excluded volume effects [27]. The constraints of conformation can induce the entropy-driven processes such as segregation of macromolecules [28,29], translocation through narrow pores [30,31] and translocation through connected chamber-pore system [32], prestretching before threading through a nanopore [33,34], etc. Investigation of Brownian polymer motions in nanoconfinement permitted development of methods for sequencing, manipulations, sorting, and separation of DNA molecules [35,36]. The shift of the ballistic/diffusive transition point towards longer times can be induced by the introduction of some geometrical constraints to space in which diffusion takes place such as slits, nanochannels, or by the limitation of diffusion to two-dimensional square lattice [37].

Anomalous diffusion has been repeatedly observed in the systems containing branched polymers [38,39]. As reported by Romiszowski and Sikorski, the longitudinal unidirectional motion of the star-branched chains can be observed in a very narrow nanochannel [40]. This motion happens when one arm points towards a particular direction, while the other two arms are left behind the branching point. The authors of References [40] have postulated a mechanism of the ballistic motion based on the fact that the two arms, located opposite to the direction of motion push the whole structure towards the direction of motion. As observed, since the nanochannel is still large enough to enable the arm’s ends to change their position passing from one side of the molecule to the other, the direction of the longitudinal motion of the chain changes chaotically. A similar anomalous diffusion governed by the active noise amplitude has been observed by Saito et al. [41].

The explanation of the longitudinal unidirectional motion of star macromolecules in tight confinement suggests that similar behavior of a wider class of asymmetric chains can be expected, e.g., even in the simple case of chains built of two parts of different properties. Therefore, the aim of this study is the examination of diffusion of a simple asymmetric chain—a block copolymer—composed of two parts of the same number of beads but of different energies of interaction between the segments. The energies were chosen so that one half of the chain could be treated as immersed in the athermal solution, and the other one was characterized by a set of interactions preferring bead–bead interaction (at the Flory coefficient *χ* = 2). The difference in the energies produces differences in the local conformational entropies and radii of gyration of both parts of the chain. This asymmetric structure is supposed to be subjected to the Brownian motion that is disturbed by a longitudinal ballistic motion similar to that described in References [40], since the reversal of the chain parts in a nanochannel is unlikely.

The paper also focuses on investigation of the effect of the width of the nanochannel and the length of the chain on its diffusive properties. 

The paper is organized as follows: In Section 2, the details of the simulation technique applied to the model representation of the polymer chain and geometry of the systems are presented. The results of a single simulation and the analysis of the evolution of geometric and thermodynamic properties of the migrating chain are gathered in Section 3.1 and Section 3.2, respectively. Section 3.3 and Section 3.4 contain the analysis of the bead and the chain mass center autocorrelation functions. The distribution of the periods between position swapping of chain parts is analyzed in Section 3.5. The geometrical aspects and thermodynamics of a single swapping event in very short time steps are presented in Section 3.6. Then, Section 3.7 presents a hypothetical explanation of the long-lasting asymmetric diffusion of the studied copolymers. A summary of the results is given in Section 3.8. 

## 2. Model and Simulation Method 

The study was performed by employing the simple and relatively fast Metropolis Monte Carlo (MC) method [42], chosen because of the ease of its implementation. As a result of the model assumptions, the studied system represents a damping dynamic model, holding only the activation aspect of diffusion and neglecting the inertia effects [43].

The simulations were performed on the three-dimensional cubic lattice of the lattice constant *b*. The confined space was a rectangular nanochannel. The length of the nanochannel was 600*b* and the width *D* was varied from 3*b* to 8*b* (depending on the simulation, in most simulations *D* = 3*b*). At both ends of the channel, the periodic boundary conditions were applied. 

The polymer chains are represented by SAWs (self-avoiding walks) embedded in the lattice. Each monomer is identified by the site on the lattice (B bead), so each chain is a sequence of *N* consecutive sites occupied by B beads. The beads succeeding along the chain are located in adjacent lattice sites at the distance *b*. The interaction energies of the type bead—bead and bead—solvent molecule and solvent molecule—solvent molecule were incorporated into the system as *ε*_PP_, *ε*_PS_, and *ε*_SS_, respectively. For simplicity, *ε*_PS_ and *ε*_SS_ were assumed to be equal to zero, whereas *ε*_PP_ was dependent on the assumed type of bead. Two different types of beads were considered: one characterized by *ε*_PP_ = 0 (i.e., immersed in the athermal solution) and the other with *ε*_PP_ not equal to zero. In most cases, *ε*_PP_ was assumed as -*k*_B_*T* corresponding to the solution of the Flory coefficient *χ* = 2 [6]. The study neglects all other intermolecular interactions, as well as interaction with nanochannel walls, except for the excluded volume effect. The studied chain consisted of *N* beads built of two equal parts: the half-chain P1 of bead type *ε*_PP_/*k*_B_*T* = 0 and the half-chain P2 of type *ε*_PP_/*k*_B_*T* = −1. P1 and P2 half-chains can be interpreted as immersed in the poor and good solvent with the tendency to form globules and loose conformation, respectively [6]. 

Fluctuations in the conformational entropy of the chains were examined by using the modified SCM (statistical counting method) [44] from the following equation:

(2) $$\frac{S}{k_{B}}=\sum_{i=1}^{N-1}\ln\left(\omega_{i}\right)$$

where *N* denotes the number of beads in the chain, *k*_B_ is the Boltzmann constant, and *ω*_i_ is the effective coordination number of each succeeding bead in the chain, which is equal to the number of lattice sites occupied by the solvent molecules. The modification consists in the application of the “phantom chain”: Initially, the whole chain was removed from the simulation space, and then—following the chain path—the *ω*_i_ value was calculated in a step-by-step manner. After each step, one succeeding bead was put back to the chain structure.

The temporary internal energy of the system was calculated as the sum of interaction energies of all current bead pairs *m* occupying the adjacent lattice sites *ε*_PP_ ≠ 0

(3) $$\frac{U}{k_{B}T}=\sum_{i=1}^{m}\varepsilon_{\mathrm{PP}}$$

The Helmholtz free energy, *A* [45], of the system was calculated as the sum of i/energetic contributions, *U,* and ii/entropic contributions, *S,* associated with the chain:

(4) A=U−TS

The chain translocation was studied by means of the Metropolis MC method [46]. The elementary micromodification applied in the simulation consisted of (i) random selection of the bead to be moved; (ii) a shift of the bead to a new position (using elementary Verdier–Stockmayer algorithms which include the kink-jump, crankshaft (two segments), reptation, or end movements [47]; (iii) checking whether the new position does not violate the topological constraints or excluded volume condition; and, finally, (iv) the Metropolis criterion. The applied reptation algorithm includes a simultaneous removal of a monomer from one end of the chain, addition of a monomer to the other end, and shift of all monomers along the chain to ensure continuity of the parts P1 and P2.

The system energy defined by Equation (2) was used to calculate the Boltzmann factor in the standard Metropolis algorithm at a constant reduced temperature *T* = 1. All types of elementary micromodifications were employed with the frequency proportional to the number of objects to which they can be applied, because such a procedure provided the correct timescale of the simulation [43,48]. 

The number of beads in the chains tested was *N* = 20 ÷ 200 (in most simulations *N* = 100). Each single simulation was a result up to 10^8^/*N* MC steps (one MC step corresponds to the number of shifts needed to give each of the beads the possibility to move once, i.e., 100 elementary macromolecule micromodifications of a macromolecule of *N* = 100). The unit of the corresponding time period, *t,* was defined as one MC cycle. Part of the results was presented as functions of elementary time *t*_E_ = *t/N*. Each simulation started with 10^5^ steps of the equilibration of conformation, using the annealing with a hyperbolic cooling schedule [49]. 

The data collected from the simulation contain positions of mass centers of the whole coil and its parts P1 and P2 and all components of free energy of the system, including internal energy and conformational entropy of the macromolecule. Moreover, on the basis of the coordinates of all beads, as well as those of the center of the coil, the autocorrelation function of the whole chain, *g*, is defined as follows:

(5) $$g\left(\Delta t\right)=\langle {\left(x\left(t\right)-x\left(t+\Delta t\right)\right)}^{2}\rangle$$

and the mean autocorrelation function of beads belonging to part P1 or P2, *g*_B_, is defined as follows:

(6) $$g_{{}_{B}}\left(\Delta t\right)=\frac{2}{N}\sum_{i=1}^{N/2}\langle {\left(x_{B,i}\left(t\right)-x_{B,i}\left(t+\Delta t\right)\right)}^{2}\rangle$$

where *x* is the position of the center of mass and *x*_B,i_ is the position of bead *i* taken from the simulation trajectory, respectively. Hence, the autocorrelation function was calculated as an average of values obtained for the whole directory and all assumed time steps, Δ*t,* which is the actual parameter of the function. The *g*_B_ function was additionally averaged over the positions of the monomers belonging to part P1 or P2. 

In the course of simulations, the mutual position of parts P1 and P2 with respect to the chain mass center (the mutual orientation parameter) was detected and stored in the variable *M*. The parameter *M* was defined as follows:

(7) $$M=\left\{ \begin{array}{ll} 1\; & \mathrm{if}\;\left|r_{P1}\right|-\left|r_{P2}\right|>\delta /2\\ -1\; & \mathrm{if}\;\left|r_{P1}\right|-\left|r_{P2}\right|<-\delta /2\\ 0\; & \mathrm{if}\;\left|\left|r_{P1}\right|-\left|r_{P2}\right|\right|<\delta /2 \end{array}\right.$$

where **r**_P1_ and **r**_P2_ are vectors identifying the Cartesian coordinates of mass centers of P1 and P2 relative to the mass center of the whole chain, respectively, and *δ* = 0.01*b*. This definition is equivalent to the following expression applicable for the chain in a tight nanopore:

(8) $$M=\left\{ \begin{array}{ll} 1\; & \mathrm{if}\;x_{P1}-x_{P2}>\delta \\ -1\; & \mathrm{if}\;x_{P1}-x_{P2}<-\delta \\ 0\; & \mathrm{if}\;\left|x_{P1}-x_{P2}\right|<\delta \end{array}\right.$$

The *M* parameter took a value equal to +1 when the center of mass of P1 part was to the right of the mass center of P2 (its *x* coordinate was higher than that of P2), −1 in the opposite situation, and 0 when the difference in positions was small. In the bulk phase, the parameter behaved similarly, but the criterion of its value was based on the distance of the mass centers of parts P1 and P2 from the mass center of the whole chain. The parameter was introduced to check if the direction of chain motion was correlated with the mutual positions of parts P1 and P2.

Forces exerted by parts of the chain, resulting from changes in their free energy, were calculated as partial derivatives:

(9) $$F={\left(\frac{\partial A}{\partial x}\right)}_{T,V}$$

where T and V denote the temperature and the volume of the system, respectively, whereas their averaged impulses in the time period Δ*t* = *t*_E_ were computed as the force integrals over the time of movement: acting on P1, P2, and at the whole chain.

(10) $$I=\frac{1}{t}\int_{t}^{t+\Delta t}Fdt=\frac{1}{t}\int_{t}^{t+\Delta t}{\left(\frac{\partial A}{\partial x}\right)}_{T,V}dt$$

The averaged impulses were computed for forces acting on part P1, part P2, and on the whole chain.

## 3. Results

### 3.1. The Chain in a Tight Nanopore

**Figure 1:**
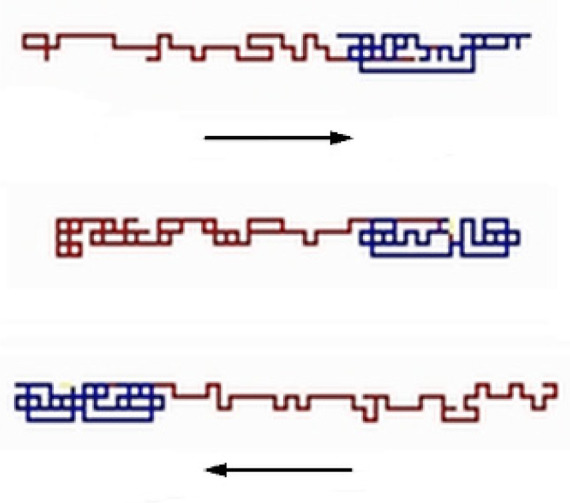
The visualization of the conformation of the macromolecule in the nanochannel. The self-attracting part of macromolecule P1 is marked in blue, whereas the athermal part P2 in red (*N* = 200, *ε*_PP_/*k*_B_*T* = −1 and 0 for P1 and P2, respectively, *D* = 4*b*). Arrows indicate the direction of movement of the whole chain.

Visualization of most often observed configurations of the studied chain in the course of evolution in the nanopore is schematically shown in Figure 1. In the course of the simulation, both parts of the chain (P1 and P2) occupied different positions along the nanopore, except during short periods when they overlapped each other during swapping of their mutual locations. The probability of both orientations of the chain parts was the same. The conformation of P1 was usually more compact than that of P2 as a result of the assumed stronger bead-to-bead interactions. 

### 3.2. Long-Time Trajectories

**Figure 2:**
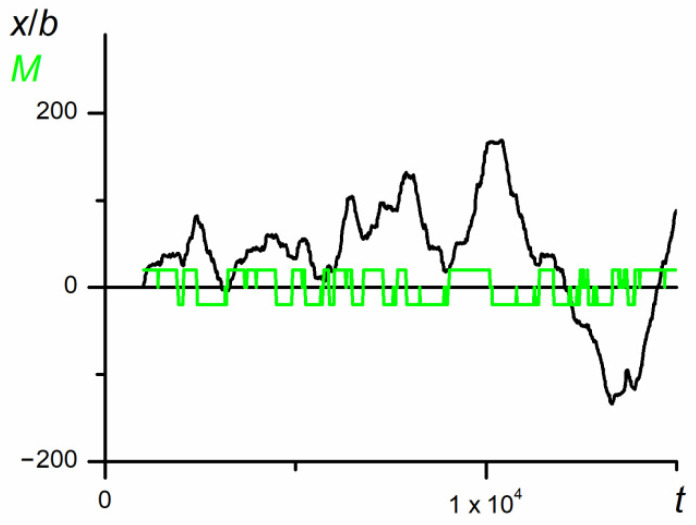
The dependence of the position of the center of mass of copolymer along the nanopore on the number of MC steps (*N* = 100, *ε*_PP_/*k*_B_*T* = −1 and 0 for P1 and P2, respectively, *D* = 3*b*, the coordinate *x* = 0 was assumed as a starting point of the fragment of trajectory shown in the Figure). The mutual orientation, *M*, along the *x*-axis is marked in green. The chart is based on the data recorded in periods of *t*_E_ = 10^3^ elementary steps.

As result from the analysis of the chain trajectory recorded for a relatively long time (10^7^*t*_E_), the studied chain showed a chaotic diffusive motion that generally was not spatially oriented. However, for shorter time periods (10^5^*t*_E_, Figure 2), the situation changed and the observed movements proved to be directed toward one or the other end of the nanopore over relatively long times. Figure 2 shows the position of the mass center of the whole chain along the nanopore and the mutual orientation parameter, *M,* as a function of *t*. As seen, the direction of the temporary unidirectional motion was correlated with the mutual positions of P1 and P2 

**Figure 3:**
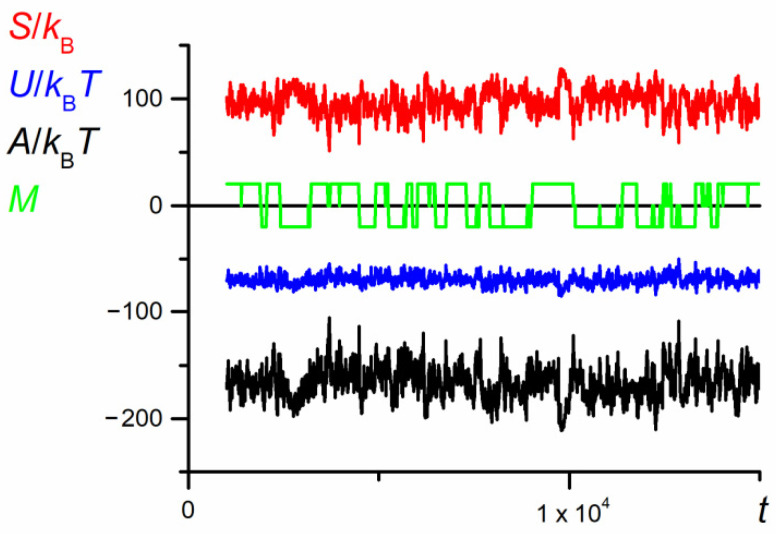
The dependence of the entropy, internal energy, and Helmholtz free energy of the chain on the number of MC steps (*N* = 100, *ε*_PP_/*k*_B_*T* = −1 and 0 for P1 and P2, respectively, *D* = 3*b*). The time changes in the mutual orientation parameter, *M,* along the *x* direction are marked in green. The chart is based on the data recorded in periods of *t*_E_ = 10^3^ elementary steps.

The swapping of P1 and P2 positions (illustrated by the change in the sign of parameter *M*) forced the change in the direction of motion in such a way that the leading part of the chain was P1, whereas athermal P2 was the following one. The results presented in Figure 2 well illustrate the unusual behavior of the chain in the nanopore. However, the results do not explain all detailed chain motions and are rather smoothed, since they are collected for long periods. In consequence, one cannot expect a direct correlation of the chain motion with its thermodynamic properties. Indeed, the values of conformational entropy, as well as internal energy change vs. the diffusion time, shown in Figure 2, seem to be completely chaotic (see Figure 3). 

**Figure 4:**
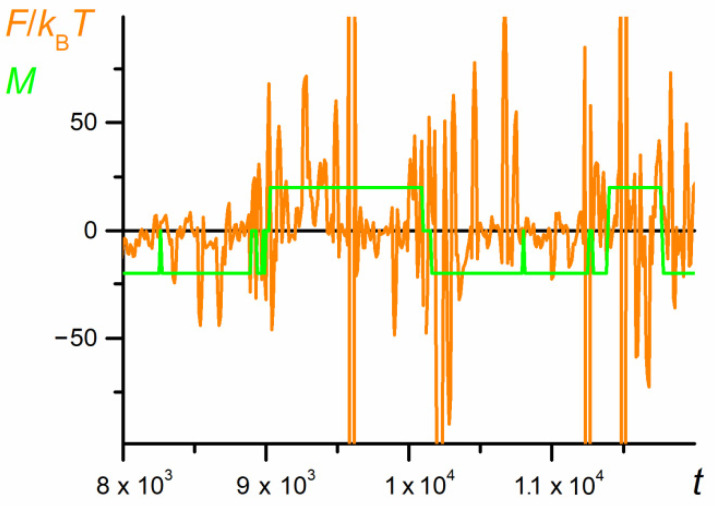
The dependence of the net force acting of the chain on the number of MC steps (fragment of the trajectory from Figure 2, *N* = 100, *ε*_PP_/*k*_B_*T* = −1 and 0 for P1 and P2, respectively, *D* = 3*b*). The mutual orientation parameter, *M,* along the *x* direction is marked in green. The chart is based on the data recorded in periods of *t*_E_ = 10^3^ elementary steps.

The dependence of the force, *F,* calculated from Equation (9), acting on the whole chain on the motion time calculated for the same data, is also chaotic (see Figure 4), and the correlation between the force and the mutual orientation parameter is elusive. 

### 3.3. Bead Autocorrelation Function

**Figure 5:**
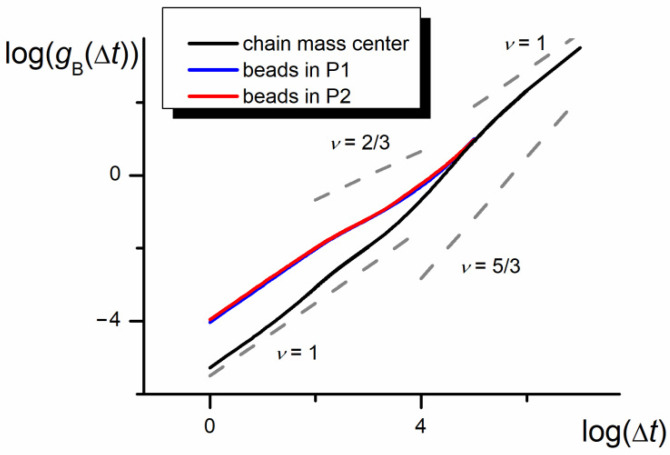
The log–log plot of the bead autocorrelation function, *g*_B_, and the chain autocorrelation function, *g,* in the nanochannel (*D* = 3*b*, *N* = 100, *ε*_PP_/*k*_B_*T* = 0, and −1 for P1 and P2, respectively). The slopes of diffusion, subdiffusion, and quasi-ballistic modes (equal 1, 2/3, and 5/3) are marked in gray.

The results gathered above point to a distinct deviation of the copolymer motion from the expectations based on the simple diffusion behavior. However, they do not provide a quantitative characterization of motion. Therefore, for a deeper analysis of the asymmetrical chain diffusion in the nanopore, the autocorrelation function for individual beads and the whole chain was recorded. The autocorrelation functions for beads located in P1 and P2 parts of the chain, *g*_B_, are shown in Figure 5. The functions were recorded for a large range of steps starting at Δ*t* = *t*_E_. The bead autocorrelation functions obtained are in line with expectations, when compared to the autocorrelation functions predicted by the Rouse and Zimm models [5,6,7]. The plot has three distinct regions: for a short and long time, the motion dynamics is diffusional (the *g*_B_ function scales with Δ*t*^1^). In the intermediate time, *g*_B_ increases to the power of 2/3. This exponent corresponding to the subdiffusion region agrees exactly with the value obtained from the Zimm model for both theta and athermal solvents [6,7] and is similar to the exponents predicted for the theta solvent in the Rouse model (*v* = 1/2) or the one-dimensional reptation dynamics (*v* = 1/4 and 1/2) [5,6]. 

The part of the whole chain autocorrelation function, *g,* for large Δ*t* overlaps the bead chain autocorrelation function *g*_B_. In the region of short times, the whole chain dynamics (represented by *g*) shows also asymptotically diffusional behavior, but, as expected, with a smaller value of the diffusion coefficient. In the range of intermediate times, the whole chain undergoes superdiffusion with a *g* vs. Δ*t* relationship scaling with the exponent 5/3. Such an unexpected result seems to be caused by the asymmetry of the chain and its long-time quasi-ballistic motion in tight confinement, impeding the inversion of chain parts. The observed effect is similar to the “ballistic motion” observed in a system of three-arm star chains in the narrow nanochannel of *D* = 3*b* [40]. However, the exponent observed here was smaller than *v* = 2 corresponding to the Newtonian uniform motion. 

### 3.4. The Mass Center Autocorrelation Function

**Figure 6:**
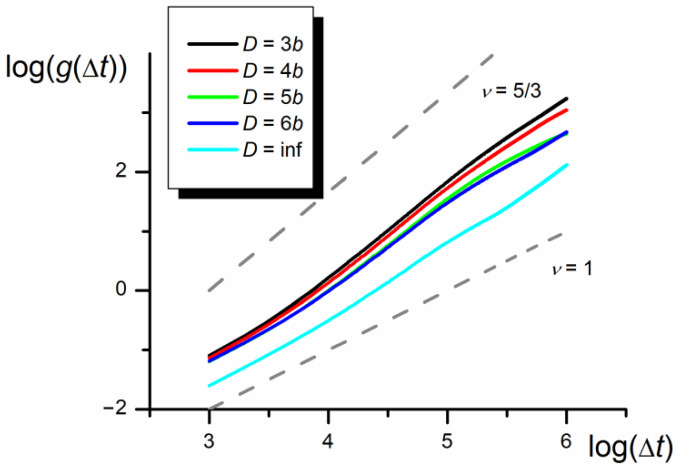
The log–log plot of the autocorrelation function, *g,* in nanochannels of different widths (*N* = 100, *ε*_PP_/*k*_B_*T* = 0 and −1 for P1 and P2, respectively). The slopes of diffusion and quasi-ballistic modes (equal 1 and 5/3) are marked in gray.

**Figure 7:**
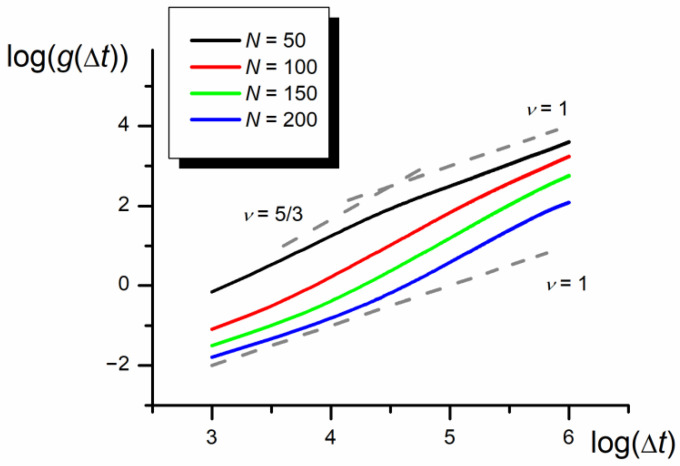
The log–log plot of the autocorrelation function, *g,* in the nanochannel for different chain lengths (*D* = 3*b*, *ε*_PP_/*k*_B_*T* = 0 and −1 for P1 and P2, respectively). The slopes of diffusion and quasi-ballistic modes (equal 1 and 5/3) are marked in gray.

**Figure 8:**
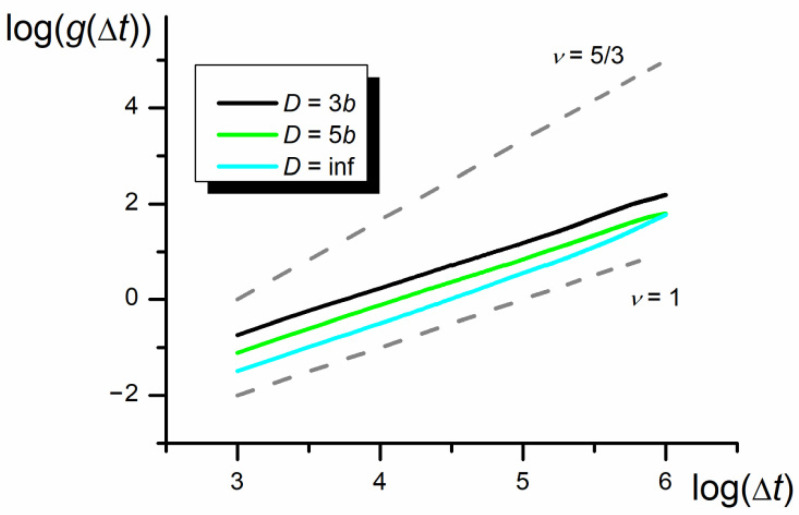
The log–log plot of the autocorrelation function, *g,* in the nanochannel and 3D space (*N* = 100, *ε*_PP_/*k*_B_*T* = 0 for P1 and P2—athermal solution). The slopes of diffusion and quasi-ballistic modes (equal 1 and 5/3) are marked in gray.

The results collected in Figure 6, Figure 7 and Figure 8 show the whole chain autocorrelation function, *g,* obtained for different chain lengths and nanopore widths. The calculated autocorrelation functions based on the shift of the chain mass center in three dimensions and along the *x* coordinate parallel to the nanopore axis, are identical with the accuracy of the standard deviation of presented results. Therefore, only the results obtained for 3D shifts are presented below. 

As shown in Figure 6, all chain motions in short and long times are diffusive with the *g* vs. Δ*t* relationship described by scaling exponent 1. In the intermediate time, the slope of the log–log plot decreases with increasing width of the nanopore and goes down starting from 5/3 for *D*/*b* = 3 to almost 1 for the chain located in 3D space without any geometrical constraints (denoted as *D* = inf). 

The slope of the *g* vs. Δ*t* dependence equal to 5/3 for *D* = 3*b* practically does not depend on the chain length (evident in the range *N* = 100–200). However, the range of the intermediate superdiffusive part of the curve shifts with the chain length increasing towards higher Δ*t* values. 

To check whether the observed phenomenon is not an artificial effect of the assumed method or model, some additional simulations for the symmetric chain wholly immersed in the athermal solvent (*ε*_PP_/*k*_B_*T* = 0 for P1 and P2) were performed. The results collected in Figure 8 show practically no superdiffusive bias in the *g* vs. Δ*t* dependence. 

Results presented in Figure 7 indicate that there are three scaling time regions of the mean autocorrelation function. The first and the third regions are similar to the monomer autocorrelation function: in the initial region where ν = 1 and the diffusion coefficient is smaller than for the monomer, and in the final region where ν is still equal to 1, but the diffusion coefficient takes the same value as for the monomers, because all monomers move with the same velocity as the whole chain. In the intermediate region, as a result of the collective motion caused by the asymmetry of the monomers in both parts of the chain, which forces the temporary unidirectional motion of the chain mass center, the *g* = f(Δ*t*) dependence scales with higher exponent than ν = 1. The *g* = f(Δ*t*) relationship for the intermediate region significantly depends on the nanopore width—the narrower the nanopore the higher the scaling exponent tending to ν = 5/3. The anomalous diffusion practically vanishes in the bulk phase. The increase in the monomer number in the chain influences the analyzed dependence only through the increase in the initial ordinate but at the same total diffusion coefficient. 

### 3.5. Time Periods between Swaps 

**Figure 9:**
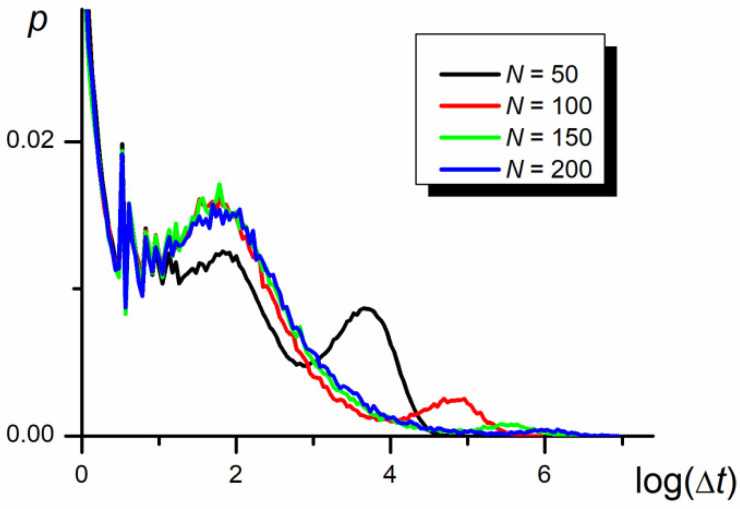
The probability density functions of periods, Δ*t*, between swapping of P1 and P2 mass centers in the logarithmic scale for different chain lengths (*t*_E_ = 5 × 10^7^, *D* = 3*b*, *ε*_PP_/*k*_B_*T* = 0 and −1 for P1 and P2, respectively).

**Figure 10:**
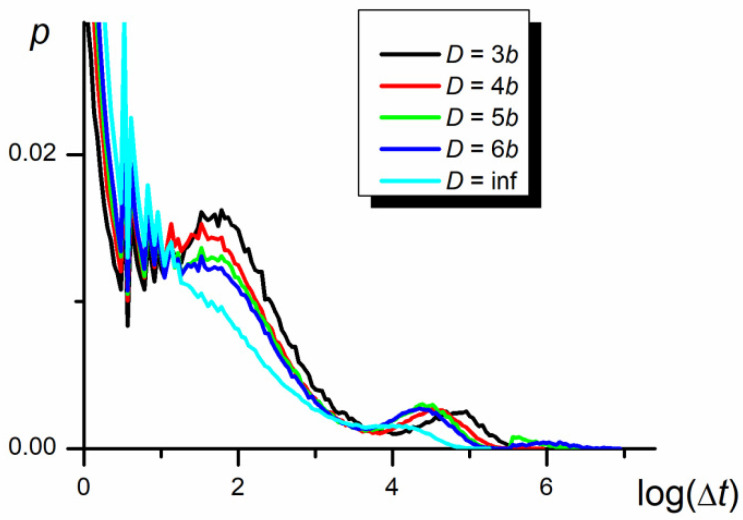
The probability density functions of periods, Δ*t*, between swapping of P1 and P2 mass centers in the logarithmic scale for different nanopore width (*N* = 100, *ε*_PP_/*k*_B_*T* = 0 and −1 for P1 and P2, respectively).

**Figure 11:**
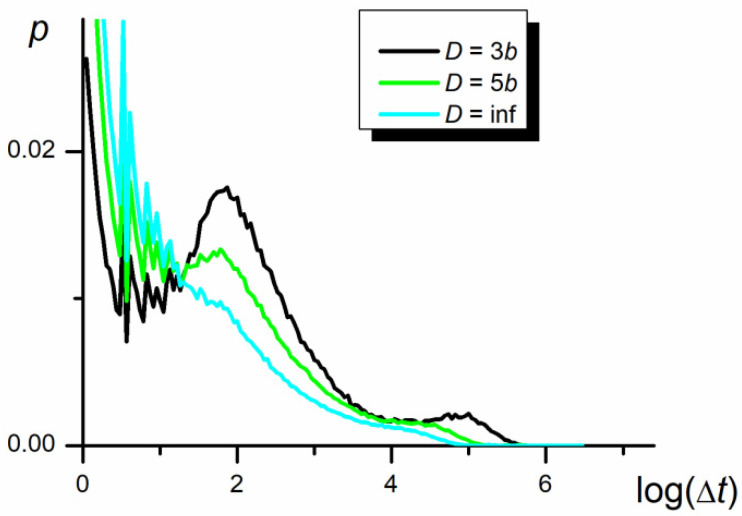
The probability density functions of time periods, Δ*t*, between swapping of P1 and P2 mass centers in the nanochannel and in the 3D space in the logarithmic scale at different nanopore width (*N* = 100, *ε*_PP_/*k*_B_*T* = 0 for P1 and P2).

Probability density functions in the time periods between the swapping of P1 and P2 positions, resulting in the inversion of the direction of chain movement, are presented in Figure 9, Figure 10 and Figure 11 in the logarithmic scale. As seen, the functions are very wide and behave as the three-modal log-normal distributions. Three maxima are evident in the case of diffusion in the narrow nanochannel. The first maximum can be interpreted as a result of the small fluctuations of P1 and P2 centers’ positions over the center of the whole chain when both parts practically overlap each other in the course of the swapping event at *M* = 0 (see Figure 2). The other two maxima seem to be produced by the mutual migration of a number of segments causing the movements of parts of the chain and of the whole chain. 

The second maximum is practically independent of the chain length (Figure 9) but slightly depends on the nanopore width (Figure 10). Since the longitudinal chain extension is larger than *D* and *D* > *b*, the chain can be represented by the de Gennes blob model, e.g., a string of self–avoiding compression blobs of diameter, *D* [25,50,51,52,53]. However, in contrast to Reference [25], reporting the relaxation time decreasing with *D* to the power −1.3, the time between swaps decreases with the nanopore width to a power of about −0.2 ± 0.2. 

The third maximum depends on the nanopore width in the way similar to that of the second one, while its position and height strongly vary with the chain length. The smaller the chain length, the higher the probability of the swap of P1 and P2 positions, which results in a smaller time between swappings and the higher number of swap events. The increase in Δ*t* caused by increasing *N* remains in a quantitative agreement with the Zimm model [6].

In summary, the three successive modes of distribution are associated with the swapping of larger and larger elements of the chain starting with the swapping caused by the move of a single monomer (the relaxation time is independent of both *N* and *D*), by the fragment of the chain of the size of blob (the relaxation time depends on *D*) and both relatively large parts P1 and P2 (the relaxation time depends on *N*). 

The distributions collected in Figure 11 show that swapping of identical parts of the chain described by the third maximum is a slightly rarer event, as compared to the swapping of parts of different interaction energies (Figure 10), but the maximum still remains. However, since the symmetry of the chain does not force the directed motion, the autocorrelation function does not detect any anomalous diffusion behavior of the chain with identical P1 and P2 parts (see Figure 8). 

### 3.6. A Single Swapping Episode

Since the prolonged movement of the chain in one direction seems to be related to the difficulty encountered by the swapping process, the trajectory, conformation, and thermodynamics of the chain were examined in the immediate surroundings of the swapping point. 

**Figure 12:**
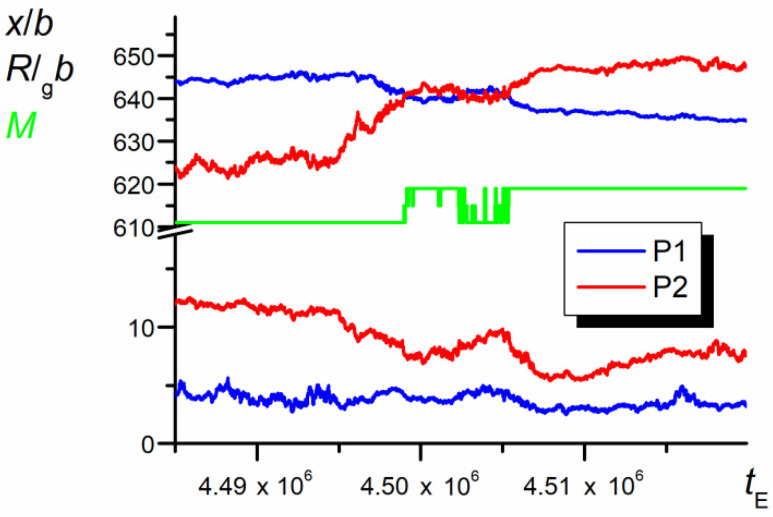
Short parts of trajectories of s P1 and P2 and their radii of gyration (*N* = 100, *ε*_PP_/*k*_B_*T* = −1 and 0 for P1 and P2, respectively, *D* = 3*b*). The chart is based on the data recorded at each elementary step, *t*_E_.

The details of the trajectory of P1 and P2 in the course of a single swap episode are shown in Figure 12. The data collected here were recorded with the time step 10^5^ (1000*N*) times smaller than that of the data shown in Figure 2. In the course of diffusive motion, after quick approach of centers of P2 to P1 (P2 is more mobile), both parts overlap each other and irregularly fluctuate around the common center of mass. Finally, P1 and P2 segregate and relax. The direction of motion of the mass center of the whole chain occurring in the same time is determined by the mutual orientation parameter *M*: Initially, the chain moves to the right, then fluctuates around a certain *x* coordinate, and finally returns back, since the swapping positions causes the inversion of direction of motion. 

The swapping of P1 and P2 positions is accompanied by the change in conformation of both parts of the chain. As seen, the size variations concern mainly P2. The approach of the centers of both parts to each other results in a decrease in *R*_g_ of P2, whereas the overcoming of P2 through P1 increases its *R*_g_. At the end of the translocation, all beads of P2 gather in a compact coil at one end of P1 (both parts collapse). Finally, P2 expands tending to the free conformation unperturbed by the other part of the chain.

**Figure 13:**
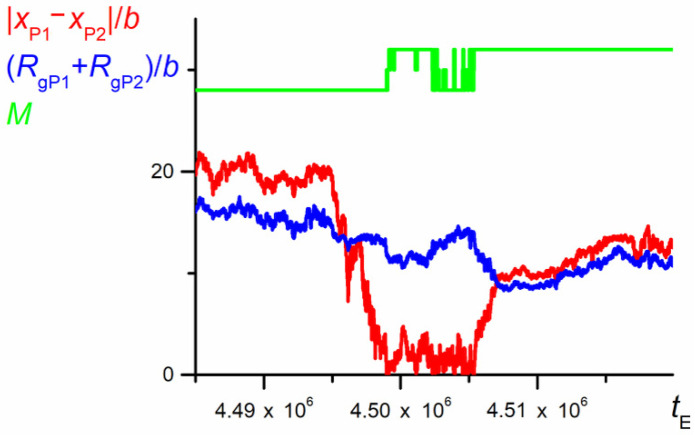
The distance between mass centers of P1 and P2 and the sum of their radii of gyration for the fragment of trajectory from Figure 12 (*N* = 100, *ε*_PP_/*k*_B_*T* = −1 and 0 for P1 and P2, respectively, *D* = 3*b*). The chart is based on the data recorded at each elementary step.

Figure 13 describes the chain conformation at a swapping event in terms of two parameters: the distance between mass centers of P1 and P2 and the sum of their radii of gyration. As shown, initially the conformation is relatively expanded—the distance between mass centers of P1 and P2 |*x*_P1_-*x*_P2_| is larger than the sum of their radii. Just before swapping, the conformation becomes more compact (decrease in *R*_gP1_ + *R*_gP2_). Then, two parts of the chain move in anti-parallel directions—during swapping, the sum of their radii of gyration slightly increases as a result of P1 and P2 elongation along the nanochannel. When the swapping ends, the sum of *R*_g_ values evidently decreases as a result of formation of compact dense coils located close to each other. Finally, both parts relax, owing to the entropic elasticity.

**Figure 14:**
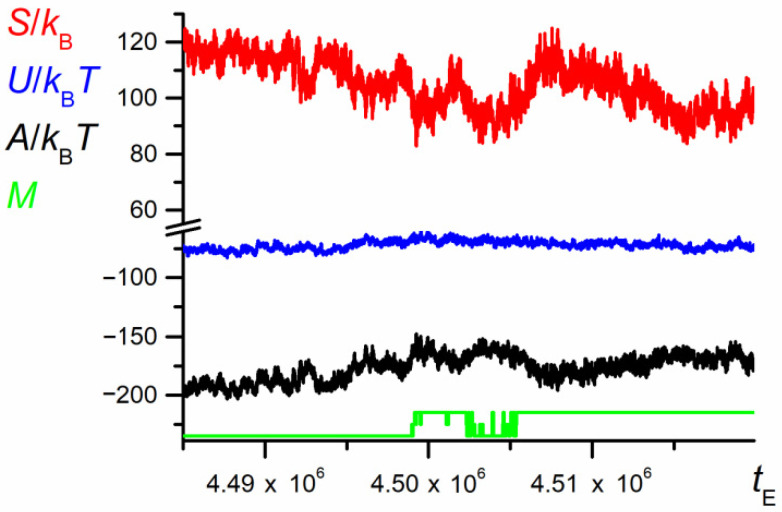
The dependence of entropy, internal energy, and Helmholtz energy of the chain on the number of elementary steps, *t*_E_, for the fragment of trajectory from Figure 12 (*N* = 100, *ε*_PP_/*k*_B_*T* = −1 and 0 for P1 and P2, respectively, *D* = 3*b*). The chart is based on the data recorded at each elementary step.

The evolution of thermodynamic properties of both chain parts (see Figure 14) is disturbed by the random noise similar to the large scale dependence shown in Figure 3. However, one can notice a small positive fluctuation of the Helmholtz energy accompanying the start of the swapping process. The mechanism of the appearance of this small Helmholtz energy barrier is entropic in nature. 

**Figure 15:**
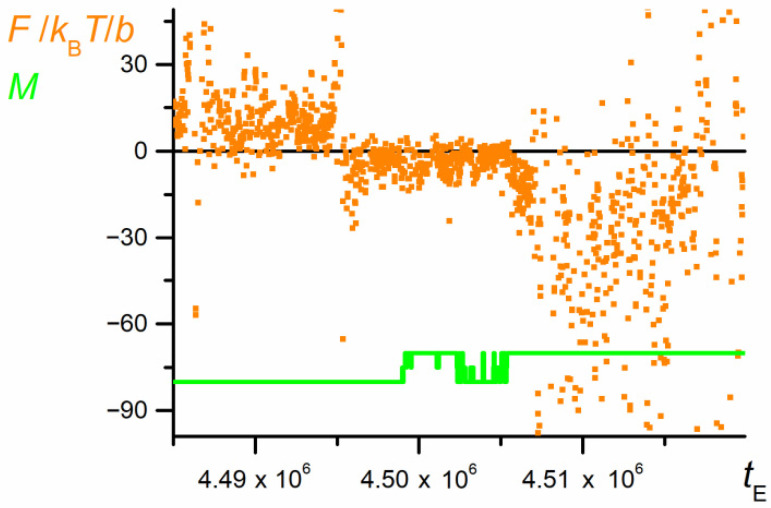
The net force caused by changes in the free energy exerted by both parts of the chain (*N* = 100, *ε*_PP_/*k*_B_*T* = −1 and 0 for P1 and P2, respectively, *D* = 3*b*). The chart is based on the data recorded at each elementary step.

The relationship *A* = f(*x*) (not presented here, but shown for a shorter part of trajectory in Figure 17 in Section 3.7) allows the calculation of force caused by the free energy fluctuations. The resulting plot shown in Figure 15 is still highly irregular, but it shows a statistically significant trend: Initially, the force takes positive values; at the moment just before the first swapping (when coils P1 and P2 start to overlap), the force is negligibly small, whereas the releasing chain produces a force highly fluctuating but of significantly negative values. Since the force is calculated as the partial derivative of the Helmholtz energy (Equation (9)), its positive values mean providing energy to the coil and its compression, whereas the negative forces correspond to the chain stretching.

The mechanical asymmetry of the properties of both parts of the chain is clearly illustrated by the impulses of forces [54] exerted by both parts of the chain. The force impulse, as an integral of the force over time, gives the information about the force cumulative contribution to the variation of the chain momentum, exerted by a certain part of the chain. The results are only qualitative, since the force impulse is not equivalent to the momentum of diffusion transfer as the system under consideration is not Newtonian. 

**Figure 16:**
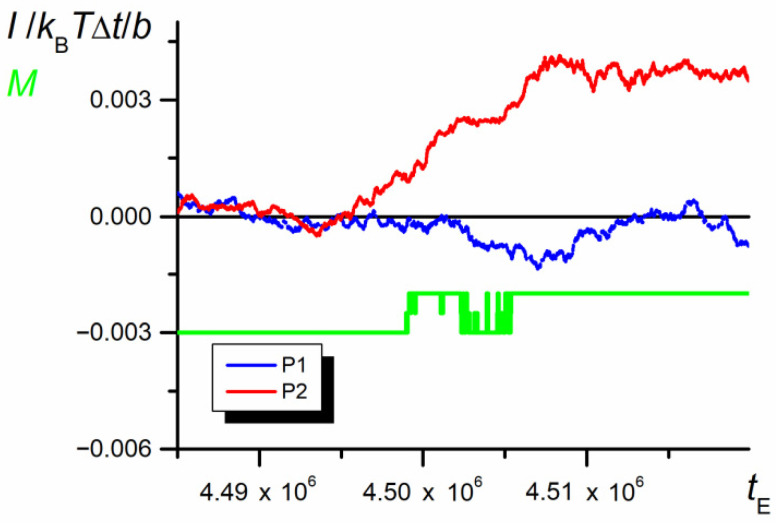
Impulses of forces caused by changes in the free energy exerted by both part P1 and P2 of the chain (*N* = 100, *ε*_PP_/*k*_B_*T* = −1 and 0 for P1 and P2, respectively, *D* = 3*b*). The chart is based on the data recorded at each elementary step.

Figure 16 shows the impulses exerted by both parts of the chain vs. time of motion. The forces were calculated on the basis of fluctuation of the Helmholtz energy of P1 and P2 separately (Equation (9)) and then integrated (Equation (10)) starting at a certain time before swapping. As shown, except for the initial steps in which the mass centers of both parts of the chain approach each other, P2 pushes P1 all the time. 

### 3.7. Mechanism of Unidirectional Motion

The results presented above allow the following description of the phenomena occurring in the studied system. The chaotic and stochastically symmetrical diffusion of beads constituting the asymmetrical chain is a collective property which produces temporal unidirectional motion of the asymmetrical chain located in a tight confinement, hindering the swapping of chain parts. The motion violates the second law of thermodynamics [45] but only for a relatively short period of time.

The phenomenon of the longitudinal motion of the asymmetrical chain represented by two sub-chains of different Flory interaction coefficients should be related to higher conformational entropy and higher diffusional mobility of the weaker interacting sub-chain P2. The P1 fragment of the chain is less mobile, as compared to P2, since instead of quasi-free bead motions in P2 limited only by the requirement of the chain continuity, its beads form aggregates which can move only in a collective way or their motion is associated with breaking of attractive interaction with one bead and creating a new one with another one. Changes in the radius of gyration of P2, as well as changes in entropy, are asymmetric in time: The decrease in both parameters is faster than their increase because some lattice nodes are blocked by P1 beads which release them relatively slowly. As a result, the fast movement is inhibited more strongly than the slow one as in the scallop effect [55]. There is no inertia effect incorporated in the model, but there are some geometrical constraints instead. In consequence, the P2 sub-chain exerts a pushing pressure on all objects around it, among others on the stopper in the nanochannel formed by the more compact and stronger interacting sub-chain P1. As a result, it produces the net force whose fluctuations averaged over a relatively long period of time are directed towards P1. Swapping is a rare event and requires overcoming of the entropic barrier. 

**Figure 17:**
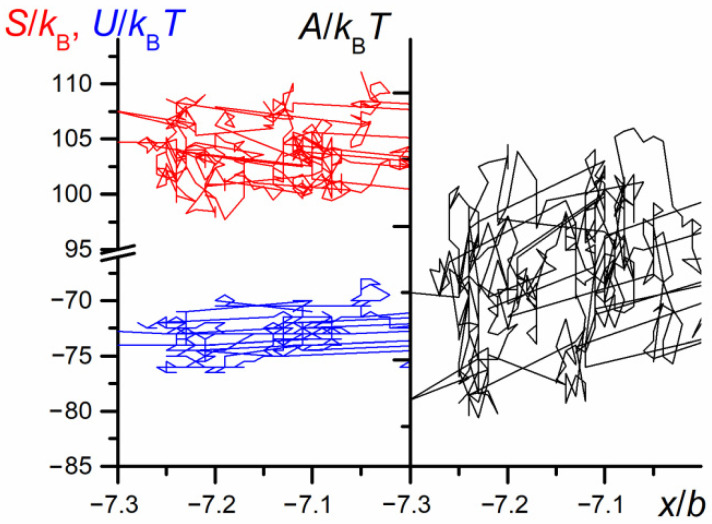
The entropy, *S*, internal energy, *U*, and Helmholtz free energy, *A*, profiles obtained from the simulation (*N* = 100, *ε*_PP_/*k*_B_*T* = −1 and 0 for P1 and P2, respectively, *D* = 3*b*). The chart is based on the data recorded at each elementary step, *t*_E_.

The trajectory mapping of the free energy changes along the diffusion trajectory, for a very small part of trajectory where only unidirectional motion occurs (*M* = 1), is shown in Figure 17. The figure demonstrates changes in entropy, internal energy, and finally free Helmholtz energy in the course of chain diffusion. As is clearly visible, the changes in the Helmholtz free energy are caused mainly by entropic effects. The Figure demonstrates that the energy vs. position of migrating species shows asymmetry of the Helmholtz free energy versus the position of the chain. The energy profile shown here reminds the figure of energy profile characteristic of behavior of the systems in which the ratchet mechanism of motion takes place [56]. 

**Figure 18:**
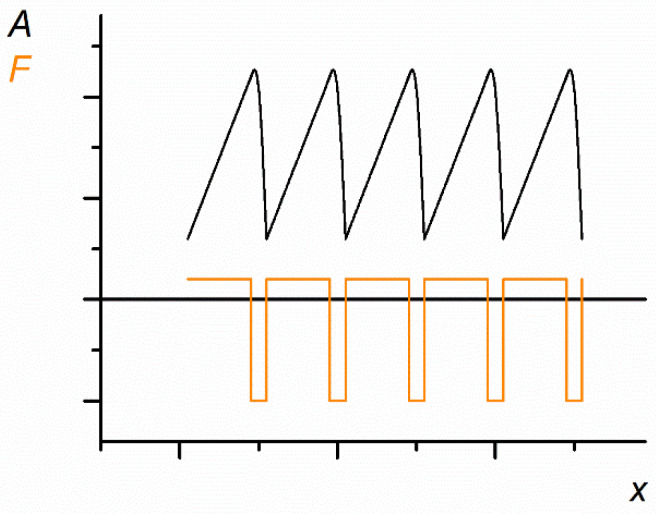
Schematic presentation of the energy and the force dependence on the position of an object moving as a result of the ratchet mechanism.

The necessary condition of the ratchet mechanism of asymmetric diffusion is the asymmetric dependence of energy on position of migrating species, as schematically shown in Figure 18. Such a profile induces asymmetry of the driving force. In consequence, the slow motion caused by a relatively small but long-lasting force is more effective than the fast one. The effect is similar to the slow-open, fast-close actuation mechanism of the scallop motion, the molecular motors-driven internal sliding of polymeric filaments in singly flagellated eukaryotes [57,58]; however, instead of the inertia or viscous effects being the cause of scallop or eukaryote motion, the motion of the analyzed copolymer is due to its energetic asymmetry. 

**Figure 19:**
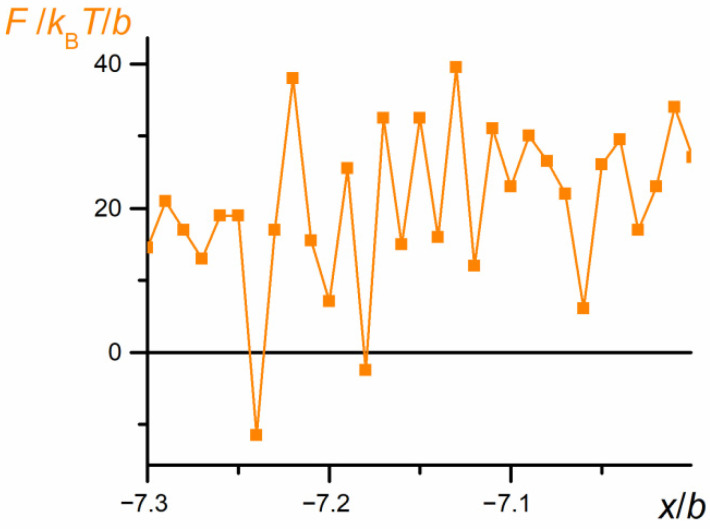
The average force, *F*, exerted by part P2 on P1 of the chain observed for a very short time period vs. the *x* coordinate of the chain mass center (*N* = 100, *ε*_PP_/*k*_B_*T* = −1 and 0 for P1 and P2, respectively, *D* = 3*b*). The chart is based on the data recorded at each elementary step, *t*_E_.

The forces exerted by part P2 on P1 of the chain computed as partial derivatives of the free Helmholtz energy (Figure 17) and averaged for positions of the chain mass center are collected in Figure 19. 

Although this force fluctuates, it is generally directed towards increasing values of the *x*-coordinate, that is, in the current direction of the chain motion. This seems to support the hypothesis of a collapsing mechanism of the chain diffusion that occurs between swapping moments. 

### 3.8. Conclusions

In solutions of the two-block copolymers, whose monomer properties differ in the interaction energies with the solvent molecules, placed in the constrained environment, namely in a tight nanopore, the anomalous diffusion of polymer is observed. The surprising behavior of the system consists in the unexpected longitudinal diffusion of molecule along the nanopore. The mechanism of the specific diffusion behavior involves two phenomena:

1. The unidirectional motion of the chain between swapping episodes. The mechanism of the unidirectional motion relies on the overcoming the asymmetric landscape of the Helmholtz free energy versus the actual chain position. This asymmetry produces the temporal impulse of force acting between active P2 and passive P1 subchains. Changes in the force impulse produce asymmetrical changes in the momentum of the chain and finally the temporal asymmetric motion of the chain.
2. The change in the direction of the diffusion motion requires overcoming of the Helmholtz free energy barrier accompanying the exchange of positions of both chain parts in confined environments. Such events are rather rare.

The anomalous diffusion of two-block copolymers depends strongly on the width of the nanopore and on the chain length. The observed abnormal diffusion is well illustrated by the anomalous behavior of the autocorrelation function of motion of the mass center of the whole chain. This particular behavior deals with the specific diffusion of the chain in the intermediate range of the autocorrelation function, where the scaling exponent takes values tending to 5/3 for very tight confinement. The probability density function of time required to swap parts of the chain is three-modal and consists of the swapping caused by the move of a single monomer, a fragment of the chain of the size of a blob, and by both parts of the two-block copolymer.

The observed dependencies can be explained, in detail, on the basis of the blob theory.
